# Menthol Inhibits Detrusor Contractility Independently of TRPM8 Activation

**DOI:** 10.1371/journal.pone.0111616

**Published:** 2014-11-06

**Authors:** Antonio Celso Saragossa Ramos-Filho, Ajay Shah, Taize Machado Augusto, Guilherme Oliveira Barbosa, Luiz Osorio Leiria, Hernandes Faustino de Carvalho, Edson Antunes, Andrew Douglas Grant

**Affiliations:** 1 Department of Pharmacology, University of Campinas (UNICAMP), Campinas, Brazil; 2 Wolfson Centre for Age-Related Diseases, King's College London, London, United Kingdom; 3 Department of Anatomy, Cellular Biology, Physiology and Biophysics, Institute of Biology, University of Campinas (UNICAMP), Campinas, Brazil; University of Southern California, United States of America

## Abstract

Agonists such as icilin and menthol can activate the cool temperature-sensitive ion channel TRPM8. However, biological responses to menthol may occur independently of TRPM8 activation. In the rodent urinary bladder, menthol facilitates the micturition reflex but inhibits muscarinic contractions of the detrusor smooth muscle. The site(s) of TRPM8 expression in the bladder are controversial. In this study we investigated the regulation of bladder contractility *in vitro* by menthol. Bladder strips from wild type and TRPM8 knockout male mice (25–30 g) were dissected free and mounted in organ baths. Isometric contractions to carbachol (1 nM–30 µM), CaCl_2_ (1 µM to 100 mM) and electrical field stimulation (EFS; 8, 16, 32 Hz) were measured. Strips from both groups contracted similarly in response to both carbachol and EFS. Menthol (300 µM) or nifedipine (1 µM) inhibited carbachol and EFS-induced contractions in both wild type and TRPM8 knockout bladder strips. Incubation with the sodium channel blocker tetrodotoxin (1 µM), replacement of extracellular sodium with the impermeant cation N-Methyl-D-Glucamine, incubation with a cocktail of potassium channel inhibitors (100 nM charybdotoxin, 1 µM apamin, 10 µM glibenclamide and 1 µM tetraethylammonium) or removal of the urothelium did not affect the inhibitory actions of menthol. Contraction to CaCl_2_ was markedly inhibited by either menthol or nifedipine. In cultured bladder smooth muscle cells, menthol or nifedipine abrogated the carbachol or KCl-induced increases in [Ca^2+^]_i_. Intravesical administration of menthol increased voiding frequency while decreasing peak voiding pressure. We conclude that menthol inhibits muscarinic bladder contractions through blockade of L-type calcium channels, independently of TRPM8 activation.

## Introduction

Overactive bladder (OAB) affects millions of people worldwide. Although first-line pharmacological interventions are efficacious [1], their unpleasant side effects have stimulated the search for novel targets to modulate bladder contractions. Many recent studies have investigated the effects of agonists and antagonists of the Transient Receptor Potential (TRP) family of ion channels on bladder function. Instillation of the TRP Melastatin-8 (TRPM8) agonist menthol into the bladder is suggested to activate bladder sensory afferents [2], whilst inhibiting muscarinic contractions of the detrusor smooth muscle (DSM) [3]. However, the dependence of these menthol effects on TRPM8 activation has only been investigated through the use of receptor antagonists [4], with no previous studies examining menthol effects in the bladders of TRPM8 knockout mice.

The nonselective cation channel TRPM8 was first identified for its activation by moderate cooling [5], [6]. It is permeable to both Na^+^ and Ca^2+^, and is activated by diverse stimuli including cool temperatures (<25°C), menthol and icilin [5], [7]. Menthol is commonly used in topical analgesic, antipruritic, and antiseptic therapies as a consequence of the sensation of cooling it produces, mediated by TRPM8 activation [8]. However, the pharmacology of menthol is complex, and it can interact with multiple targets independently of TRPM8 [9], [10], [11].

The precise sites of expression and functions of TRPM8 in the bladder remain unclear. In human bladder samples, TRPM8 mRNA was detected in the urothelium but not in the DSM layer [12]. TRPM8 immunoreactive nerve fibres, both unmyelinated and myelinated, were identified in the suburothelium of human bladder biopsies, with immunoreactivity also in urothelial cells [13]. The density of innervation correlated well with pain and urinary frequency in patients with overactive bladder or painful bladder syndrome [13]. Menthol and icilin both produced inwards currents and an increase in cytosolic Ca^2+^ in a proportion of cultured rat urothelial cells [14]. However, in cultured mouse urothelial cells TRPM8 mRNA was below the detection level, and no TRPM8 currents or increase in cytosolic Ca^2+^ were observed following exposure of the cells to menthol [15].

TRPM8 agonists are also able to alter bladder function. Intravesical menthol administration increased micturition pressure and decreased the volume threshold for micturition in anaesthetized guinea pigs, suggesting that it activates C-fibres [16]. Intravesical menthol also decreased the volume threshold for micturition in rats, with no change in micturition pressure [3]. In the same study, incubation with menthol inhibited carbachol-induced contractions of isolated bladder strips [3]. In pig detrusor and mucosal strips, both menthol and icilin inhibited carbachol-induced contractions [17]. Moreover the TRPM8 antagonist AMTB attenuated the rat bladder micturition reflex to filling, an effect attributed to its actions on the afferent innervation [18].

It has been suggested that some of the biological effects of menthol are due to mechanisms independent of TRPM8 activation, possibly through an inhibition of voltage-depedent calcium influx [9]. Additionally, it has been proposed that menthol inhibits nicotinic receptors [10] and voltage-gated sodium channels [11] in cultured sensory neurons. In this study we investigated the modulation of muscarinic bladder strip contractions by menthol, and investigated its underlying molecular mechanisms.

## Materials and Methods

### Animals

TRPM8 wild type (+/+) and knockout (−/−) mice were bred in house at King's College London. C57BL/6J mice were bred and housed at the University of Campinas, Brazil. Male mice (25–30 g) aged at least 2 months were used in these studies. All animals were maintained on normal diet, with free access to food and water, on a 12 h/12 h light/dark cycle, in a climate-controlled environment. All procedures were carried out in accordance with the UK Animals (Scientific Procedures) Act, 1986, UK and Brazilian College for Animal Experimentation (COBEA) guidance. They were approved by the Animal Welfare and Ethical Review Body at KCL and by the Committee for Ethics in Animal Research (CEEA-UNICAMP, protocol number 3313-1) at UNICAMP. Mouse tissues and cells used in this study were collected from animals euthanased with pentobarbital.

### Tissue preparation and organ bath set-up

Mice were euthanized with an overdose of sodium pentobarbital (1 mg/g). The bladder dome was removed above the level of the ureters, and cut into 2 longitudinal strips. Unless otherwise stated, experiments were performed on bladder strips with intact mucosa. These were suspended in a 10 mL organ bath containing Krebs' solution (117 mM NaCl, 4.7 mM KCl, 2.5 mM CaCl_2_, 1.2 mM MgSO_4_, 1.2 mM KH_2_PO_4_, 24.8 mM NaHCO_3_ and 11 mM glucose, pH 7.4) at 37°C and bubbled with a mixture of 95% O_2_ and 5% CO_2_. Changes in isometric force were recorded using a Power Lab v.4 system (AD Instruments, UK). The resting tension was adjusted to 5 mN at the beginning of the experiments. The equilibration period was 60 min, during which the Krebs' solution was replaced every 20 min.

### Electrical-field stimulation-induced bladder strip contractions

Prior to pharmacological stimulation, electrical-field stimulation (EFS) was performed with parallel electrodes positioned on either side of the bladder strips. Contractile responses to EFS were obtained at frequencies of 8, 16 and 32 Hz. The tissues were stimulated for 10 s with pulses of 1 ms width at 80 V, with 3 min intervals between stimulations. Following carbachol additions (described below), the tissues were incubated with menthol (30 or 300 µM), icilin (1 µM), nifedipine (1 µM) or vehicle (0.3% DMSO) for 15 min. The tissue was again electrically stimulated at 8, 16 and 32 Hz. Frequency-response data was also obtained in the presence of the voltage-gated sodium channel blocker, tetrodotoxin (1 µM, 30 min) to confirm the neurogenic nature of the contractions.

### Concentration-response curves to carbachol

Following EFS, cumulative concentration-response curves to the muscarinic agonist carbachol (1 nM - 30 µM) were constructed in one-half log unit increments. After the first full curve was constructed, menthol (30 or 300 µM), icilin (1 µM), the neuronal Na^+^ channel blocker tetrodotoxin (TTX; 1 µM), nifedipine (1 µM) or vehicle (0.3% DMSO) were added to the organ baths and allowed to incubate for 15 min. A further cumulative concentration-response curve to carbachol was then constructed. The participation of sodium flux in the response to menthol was also investigated by replacing the sodium chloride and sodium bicarbonate in the Krebs' solution with the impermeant cation N-Methyl-D-glucamine (NMDG) and buffer HEPES, in equimolar concentrations. In a further series of experiments, TRPM8 +/+ urothelium denuded bladder strips were obtained by using ophthalmic scissors with the aid of a stereomicroscope. Denuded bladder strips were then mounted in the organ baths, and the above was repeated.

### Pharmacological evaluation of contraction to extracellular calcium influx

Cumulative concentration response curves to CaCl_2_ (1 µM to 100 mM) using one log unit increments in depolarizing conditions were constructed, as previously described [19]. Briefly, the strips were prepared and mounted in 10 mL organ baths containing Ca^2+^-free Krebs' solution with EGTA (1 mM) to sequester Ca^2+^ ions. Preparations were contracted with carbachol (10 µM) to deplete intracellular Ca^2+^. The initial bathing solution was removed and replaced by Ca^2+^-free Krebs' solution containing KCl (80 mM) with cyclopiazonic acid (CPA; 10 µM) to block sarcoplasmic reticulum Ca^2+^ stores. After an equilibration period of 30 min, the cumulative concentration response curve to CaCl_2_ was obtained in the absence or presence of nifedipine (1 µM) or menthol (300 µM). Each strip preparation was used to construct one CaCl_2_ concentration–response curve.

### Reversal of KCl contraction of bladder strips by the addition of menthol

Following pre-contraction of the tissue strips with KCl (40 mM), a cumulative concentration–relaxation curve to menthol (10 nM–1 mM, one log unit increments) was constructed in the absence or presence of a cocktail of potassium channel blockers (charybdotoxin 100 nM, apamin 1 µM, glibenclamide 10 µM and tetraethylammonium 1 µM).

### Primary Culture of DSM Cells and [Ca^2+^]_i_ determination

Five bladders were dissected from six weeks old C57BL/6J mice and cut into sections in Hank's Balanced Salt solution (HBSS; Sigma Aldrich) containing 2% penicillin (10,000 units/mL) and streptomycin (10 mg/mL). The tissue was then incubated in RPMI1640 containing 1 mg/mL collagenase type I from *Clostridium histolyticum* (Gibco), 2% penicillin (10,000 units/mL) and 10 mg/mL streptomycin under constant stirring for 4 hours at 37°C. The resultant solution was centrifuged at 479 g and the pellet resuspended in 1 mL of trypsin/EDTA solution (Sigma Aldrich) for 5 min at 37°C. Trypsin was inactivated by adding 2 mL of RPMI1640 with 10% of fetal bovine serum. Dissociated bladder cells were counted, and 2×10^5^ cells were plated in Petri dishes (CellView, 35 mm, four compartments, glass bottom, Greiner Bio-one) containing complete medium (RPMI1640, 10% fetal bovine serum, 2% penicillin/streptomycin and 0.14% insulin), then maintained at 37°C. The culture medium was changed every 48 hours. All the experiments were done in the first cell passage. The smooth muscle phenotype of these cells was confirmed immunocytochemically by their expression of smooth muscle myosin heavy chain.

The intracellular Ca^2+^ concentration was assessed as previously described [20]. Briefly, once bladder smooth muscle cells (SMCs) achieved about 60–70% confluence, they were washed two times in HBSS (Sigma Aldrich). They were incubated in HBSS with EGTA (1 mM) and carbachol (10 µM) for 10 min at 37°C. Next, cells were washed in HBSS, loaded with 10 µM of the Ca^2+^-sensitive dye FluoForte-AM (Enzo Life Sciences, Inc., USA) and incubated with 10 µM CPA for 45 min at 37°C. Over the last 25 min menthol (30 and 300 µM), nifedipine (1 µM) or vehicle (DMSO 0.1%) were added to the medium. Changes in [Ca^2+^]_i_ were monitored in real time using a confocal microscope (Zeiss LSM780-NLO, Carl Zeiss AG, Germany). Immediately before the beginning of fluorescence recording, CaCl_2_ (1 mM) was added to the HBSS. The fluorescence of individual cells was recorded at excitation 490 nm and emission 520 nm for 2–4 minutes, when the calcium influx was stimulated with addition of KCl (40 mM), or carbachol (CCh; 10 µM) to the medium. [Ca^2+^]_i_ was expressed as (F-Fmin)/(Fmax-Fmin), the fluorescence intensity normalized to the individual maximal fluorescence for each cell. 30 cells per image were randomly chosen and defined as responding if (F-Fmin)/(Fmax-Fmin) exceeded 0.1 during the application of carbachol/KCl.

### Cystometry

Mice were anaesthetized with an intraperitoneal injection of urethane (1.8 g.kg-1). Once surgical anesthesia was reached, a 1 cm incision was made along the midline of the abdomen. The bladder was exposed and a butterfly cannula (25 G) was inserted into the bladder dome. The cannula was connected to a three-way tap, one port of which was connected to a pressure transducer and the other to the infusion pump through a catheter (PE50). Before starting the cystometry, the bladder was emptied via the third port.

Continuous cystometry (CMGs) was carried out by infusing saline into the bladder for 30–35 min at a rate of 0.6 ml.h^−1^. After completion of the first CMG, the pump was stopped and the bladder was emptied. A further cystometric recording was then carried out with instillation of menthol (30 µM or 300 µM). The following parameters were assessed: Pressure threshold (the intravesical pressure immediately before micturition); Voiding pressure (the peak pressure reached during micturition); Capacity (the volume of saline needed to induce the first micturition); Voiding frequency (the number of voids per minute).

### Drugs and reagents

Apamin, carbachol, charybdotoxin, cyclopiazonic acid, DMSO (dimethyl sulphoxide), EGTA (ethylene glycol-bis (2-amino-ethylether)- N,N,N′,N′-tetra-acetic acid), glibenclamide, (-)-menthol, NMDG (*N*-Methyl-D-glucamine), tetraethylammonium and tetrodotoxin were obtained from Sigma Aldrich, UK or Sigma Aldrich, USA. All drugs were dissolved and administered in Krebs' solution.

### Analysis of data

Nonlinear regression analysis was performed to determine the pEC_50_ using GraphPad Prism (GraphPad Software, San Diego, CA, USA) with the constraint that Φ = 0. All concentration–response data were evaluated for a fit to a logistic function in the form: 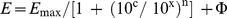
where E_max_ is the maximal response produced by agonists; c is the logarithm of the EC_50_, the concentration of drug that produces a half-maximal response; x is the logarithm of the concentration of the drug; n, the exponential term, is a curve-fitting parameter that defines the slope of the concentration– response line and Φ is the response observed in the absence of added drug.

Data are expressed as mean ± SEM of n experiments. The cumulative concentration and frequency-response data are expressed as mean of the contraction in mN mg^−1^ of wet strip weight ± SEM of n experiments to control for variances in bladder strip size. Cumulative concentration–response curves to CaCl_2_ are expressed as percentage of contractions to 10 µM carbachol. Instat (GraphPad Software, San Diego, CA, USA) was used for statistical analysis. One-way Analysis of Variance (ANOVA) followed by Tukey's test was used in all groups, and p<0.05 was accepted as significant.

## Results

### EFS-induced contractions are inhibited by menthol in both wild type and TRPM8 knockout bladder strips

**Figure 1:**
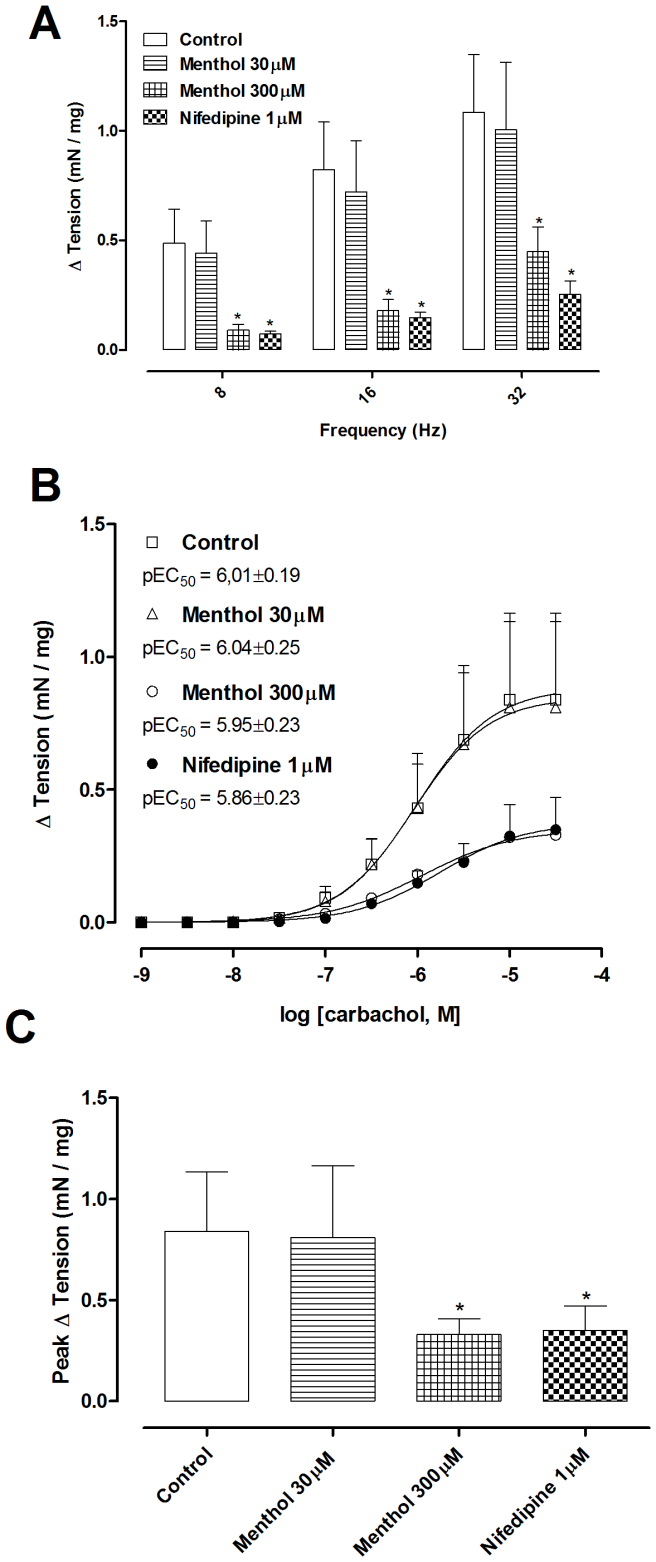
Electrical field stimulation (EFS; 8, 16 and 32 Hz; 80 V, 1 msec pulses) and carbachol (1 nM–30 µM) induced contractions of bladder strips that were inhibited by 300 µM menthol or 1 µM nifedipine. (A) The effect of incubation with menthol (15 min; 30 µM or 300 µM) or nifedipine (15 min; 1 µM) on EFS-induced contractions. (B) The effect of incubation with menthol (15 min; 30 µM or 300 µM) or nifedipine (15 min; 1 µM) on carbachol-induced contractions. (C) Comparison of maximal responses (E_max_) to carbachol in the experimental groups. *  =  P<0.05 compared with control group (one-way ANOVA followed by Tukey's post-test).

**Figure 2:**
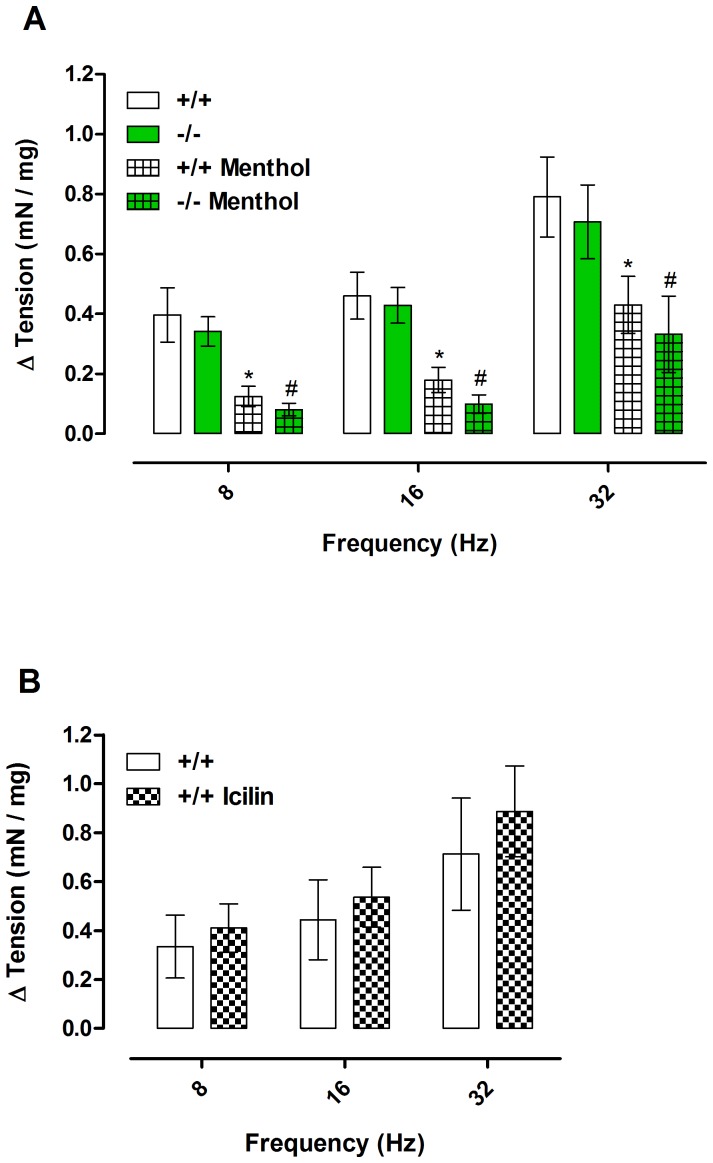
Electrical field stimulation (EFS)-induced contractions (8, 16 and 32 Hz; 80 V; 1 msec pulses) of bladder strips from TRPM8 +/+ and −/− mice in the absence or presence of TRPM8 agonists. (A) The effect of incubation with menthol (15 min; 300 µM) on EFS-induced contractions. (B) The effect of incubation with icilin (15 min; 1 µM) on EFS-induced contractions. Data represents the mean ± S.E.M. for 5–7 strips in each group. *  =  P<0.05 compared with untreated +/+; #  =  P<0.05 compared with untreated −/− (one-way ANOVA followed by Tukey's post-test).

EFS (8–32 Hz) elicited bladder contractions in bladder strips, and these contractions were unaffected by preincubation for 15 minutes with 30 µM menthol. In contrast, preincubation with 300 µM menthol or 1 µM nifedipine significantly inhibited contractions (Figure 1A). Contractions to EFS were of similar magnitude in bladder strips from TRPM8 −/− mice (Figure 2A). Incubation for 15 minutes with menthol (300 µM) significantly decreased EFS-induced contractions at all frequencies tested in bladder strips of both genotypes (Figure 2A; *P*<0.05). Incubation with icilin (1 µM, 15 min) had no effect on the contractions to EFS (Figure 2B).

### Muscarinic contractions are inhibited by menthol in both wild type and TRPM8 knockout bladder strips

**Figure 3:**
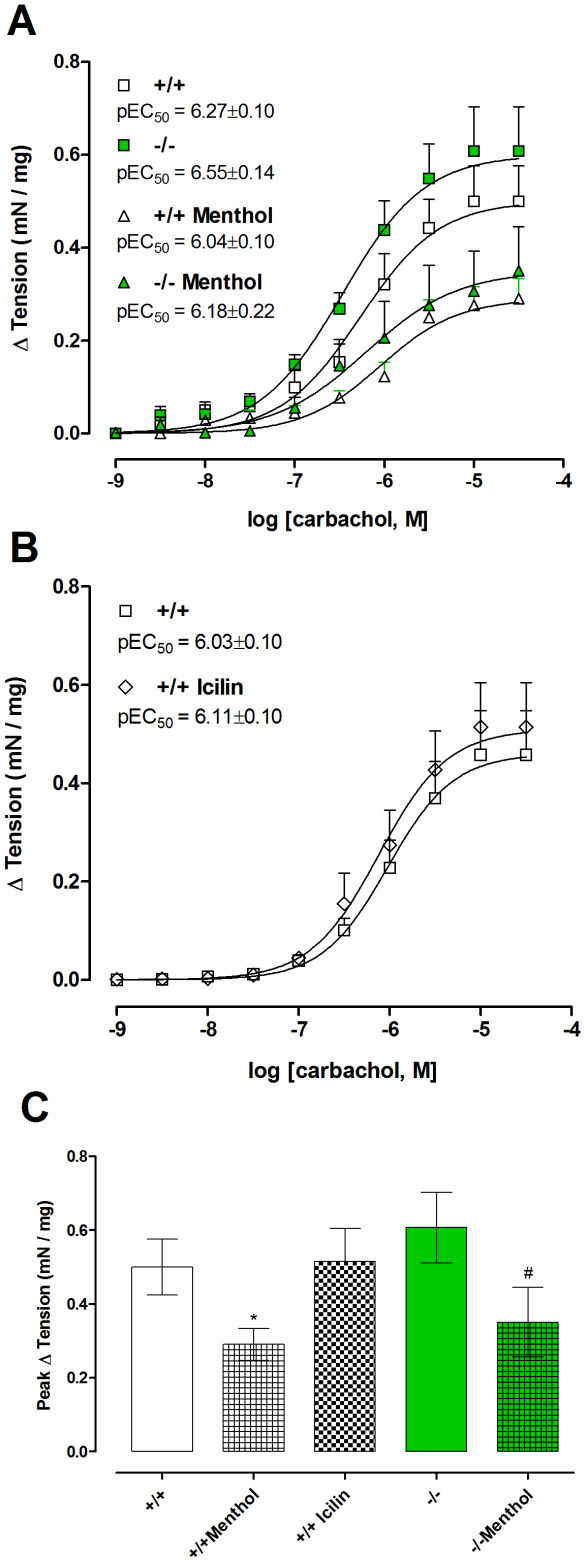
Carbachol (1 nM–30 µM) induced concentration-dependent contractions in isolated bladder strips from TRPM8 +/+ and −/− mice in the absence or presence of TRPM8 agonists. (A) The effect of incubation with menthol (15 min; 300 µM) on carbachol-induced contractions. (B) The effect of incubation with icilin (15 min; 1 µM). (C) Comparison of maximal responses (E_max_) to carbachol in the experimental groups. Data represents the mean ± S.E.M. for 5–7 strips in each group. *  =  P<0.05 compared with untreated +/+; #  =  P<0.05 compared with untreated −/− (one-way ANOVA followed by Tukey's test).

Carbachol (1 nM–30 µM) produced concentration-dependent contractions in bladder strips, and these contractions were unaffected by preincubation for 15 minutes with 30 µM menthol. In contrast, preincubation with 300 µM menthol or 1 µM nifedipine significantly inhibited contractions (Figures 1B and 1C). Contractions to carbachol were of similar magnitude in bladder strips from TRPM8 −/− mice, with no significant differences in E_max_ or pEC_50_. Incubation with menthol (300 µM, 15 min) decreased E_max_ in both groups (Figure 3A and 3C), whilst incubation with icilin (1 µM, 15 min) did not change any pharmacological parameter (Figure 3B). Incubation with the vehicle control DMSO (0.3%) did not affect the E_max_ of the carbachol-induced contractions (Basal: 0.69±0.11 mN/mg vs DMSO-treated: 0.63±0.13 mN/mg).

### Menthol inhibition of carbachol contractions is independent of the urothelium

**Figure 4:**
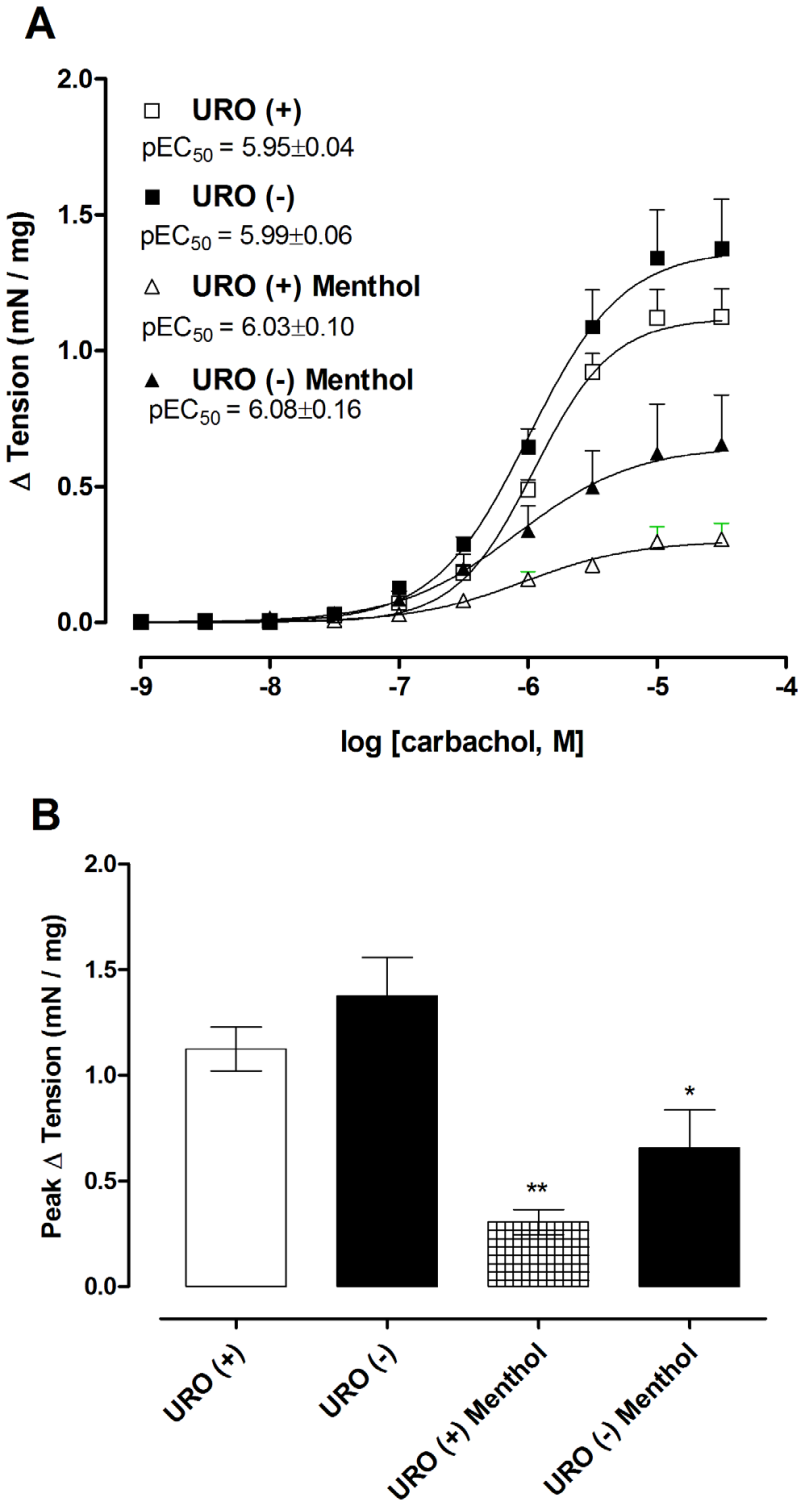
Effect of urothelial denudation on inhibition by menthol of carbachol-induced contraction of mouse bladder strips. (**A**) Concentration-response curves for carbachol (1 nM–30 µM) contractions in mucosal-denuded, URO (−), and mucosal-intact, URO (+) bladder strips, before and after incubation with menthol (15 min; 300 µM). (**B**) Comparison of maximal responses (E_max_) to carbachol in the experimental groups. Data represents the mean ± S.E.M. for 5–7 strips in each group. *  =  P<0.05 compared with untreated URO(−); **  =  P<0.01 compared with untreated URO (+) (one-way ANOVA followed by Tukey's test).

Carbachol produced similar concentration-dependent contractions in both intact and urothelium-denuded preparations (Figures 4A and 4B). Pre-treatment with menthol (300 µM) significantly inhibited contractions in both preparations (P<0.05).

### Menthol inhibition of carbachol contractions is independent of neuronal activation

**Figure 5:**
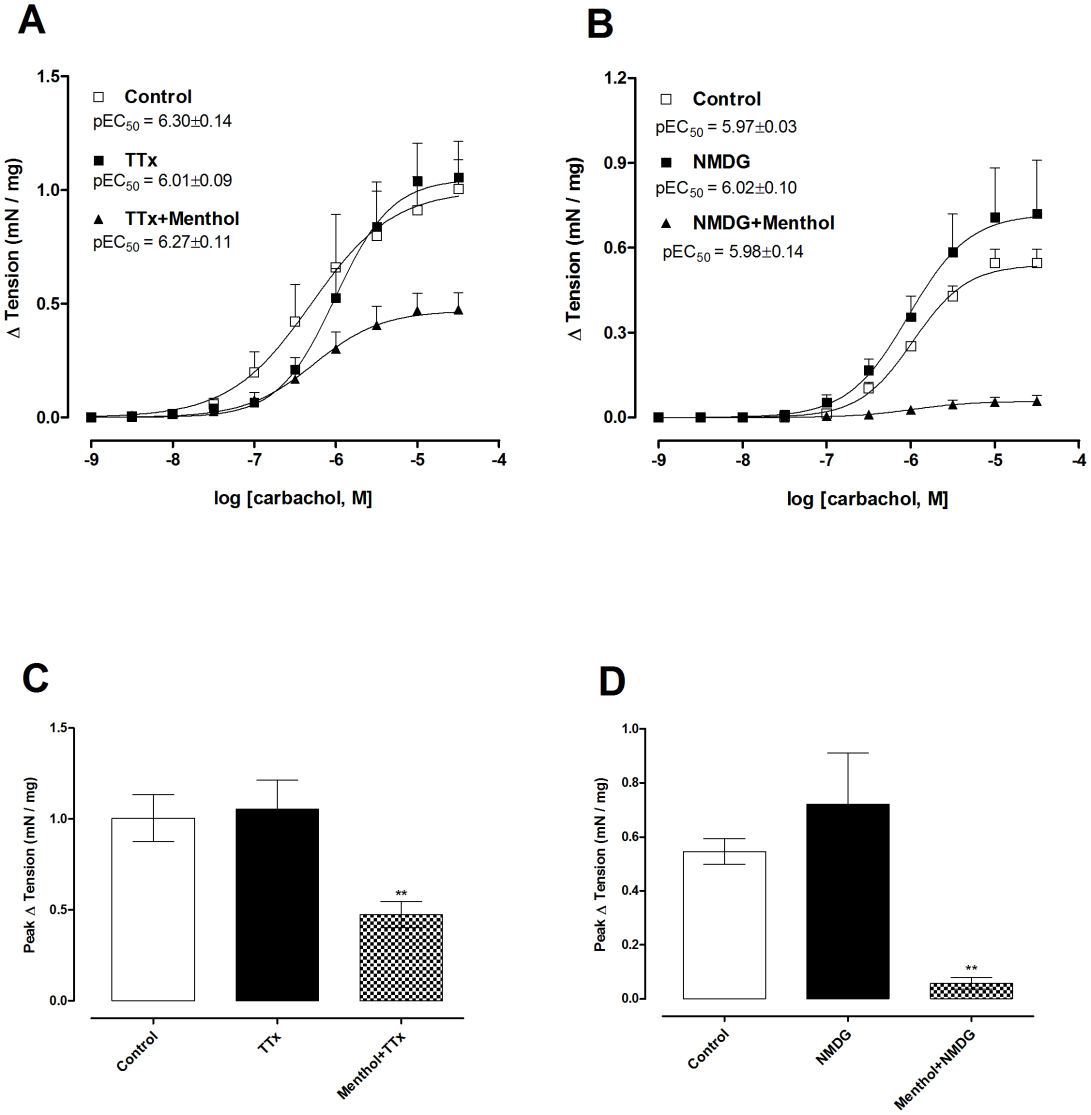
Disruption of Na^+^ channel currents does not affect menthol inhibition of muscarinic contractions. (**A**) Concentration-response curves for carbachol contractions in the presence of vehicle, TTX (1 µM) alone or TTX with menthol (300 µM). (**B**) Concentration-response curves for carbachol contractions in normal Krebs' solution, or following replacement of extracellular sodium with NMDG or NMDG with menthol (300 µM). (**C**) and (**D**) Comparison of maximal responses (E_max_) to carbachol in the experimental groups. Data represents the mean ± S.E.M. for 3–4 strips in each group. **  =  P<0.01 compared with control (one-way ANOVA followed by Tukey's test).

Neither incubation with the sodium channel inhibitor TTX (1 µM) nor replacement of the NaCl and NaHCO_3_ in the Krebs' buffer with the impermeant cation NMDG (130 mM) and HEPES (25 mM) affected the contractions produced by carbachol (Figure 5). However, incubation of the bladder strips with TTX or NMDG/HEPES buffer combined with menthol (300 µM) significantly inhibited carbachol-induced contraction (P<0.01) (Figure 5).

### Menthol relaxation of KCl-precontracted bladder strips is independent of potassium channel activation

**Figure 6:**
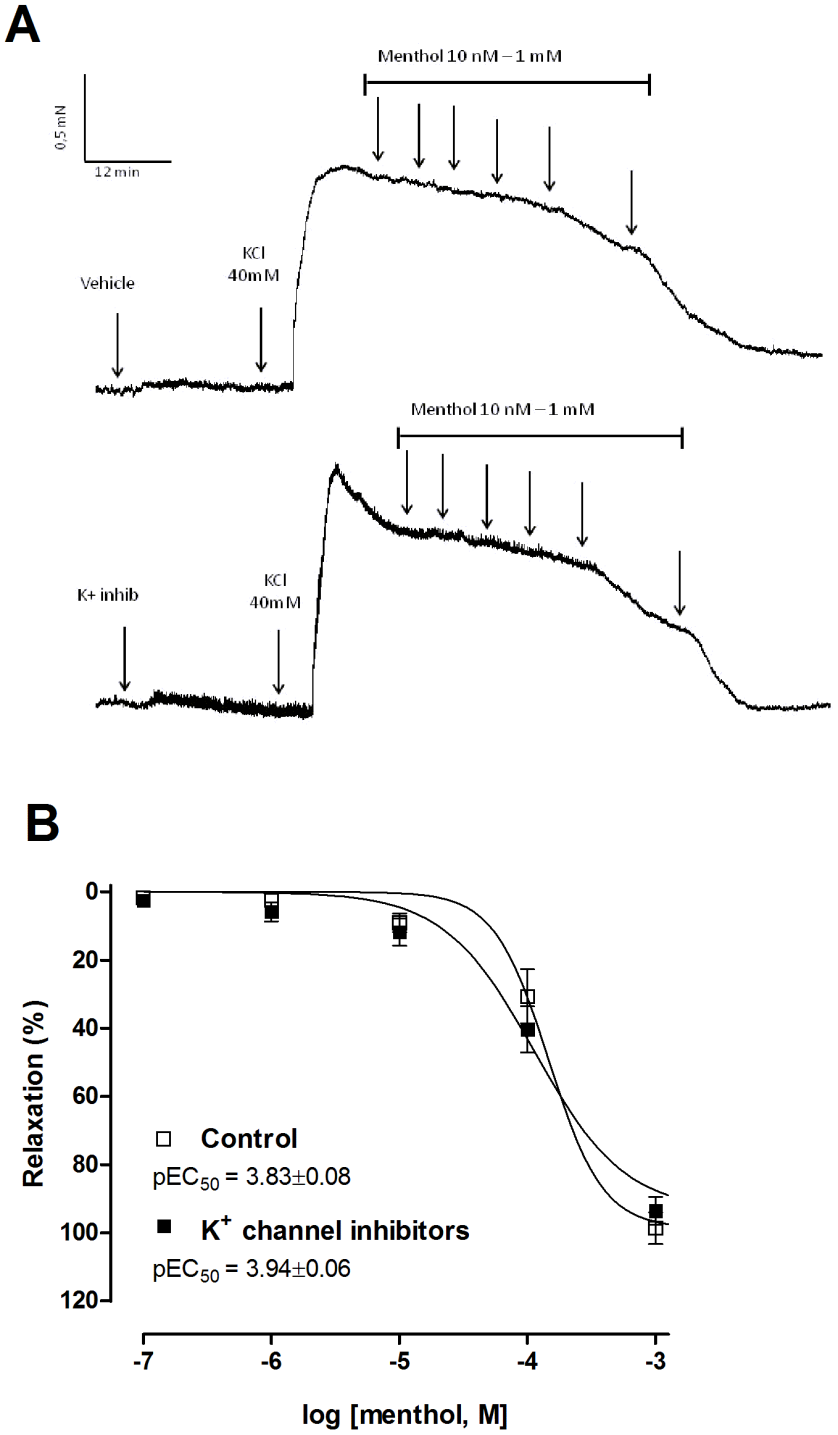
Blockade of K^+^ channels does not affect relaxation of KCl-precontracted bladder strips. (**A**) Original tension recordings illustrating concentration-response relaxing curves for menthol (10 nM–1 mM) in pre-contracted bladder strips (KCl 40 mM) with or without cocktail of K^+^ channel blockers pre-treatment (20 min; 100 nM charybdotoxin, 1 µM apamin, 10 µM glibenclamide and 1 µM tetraethylammonium). (**B**) Summary data showing no statistical changes between menthol-curves in the absence and presence of K+ channels blockers. Data represents the mean ± S.E.M. for 4 strips in each group.

Menthol (10 nM–1 mM) induced a concentration-dependent relaxation of bladder strip contractions produced by 40 mM KCl (Figure 6). Pre-treatment with a cocktail of K^+^ channel inhibitors (100 nM charybdotoxin, 1 µM apamin, 10 µM glibenclamide and 1 µM tetraethylammonium) had no effect on the relaxation produced by menthol (Figure 6).

### Menthol and nifedipine both inhibit bladder strip contractions due to extracellular calcium influx

**Figure 7:**
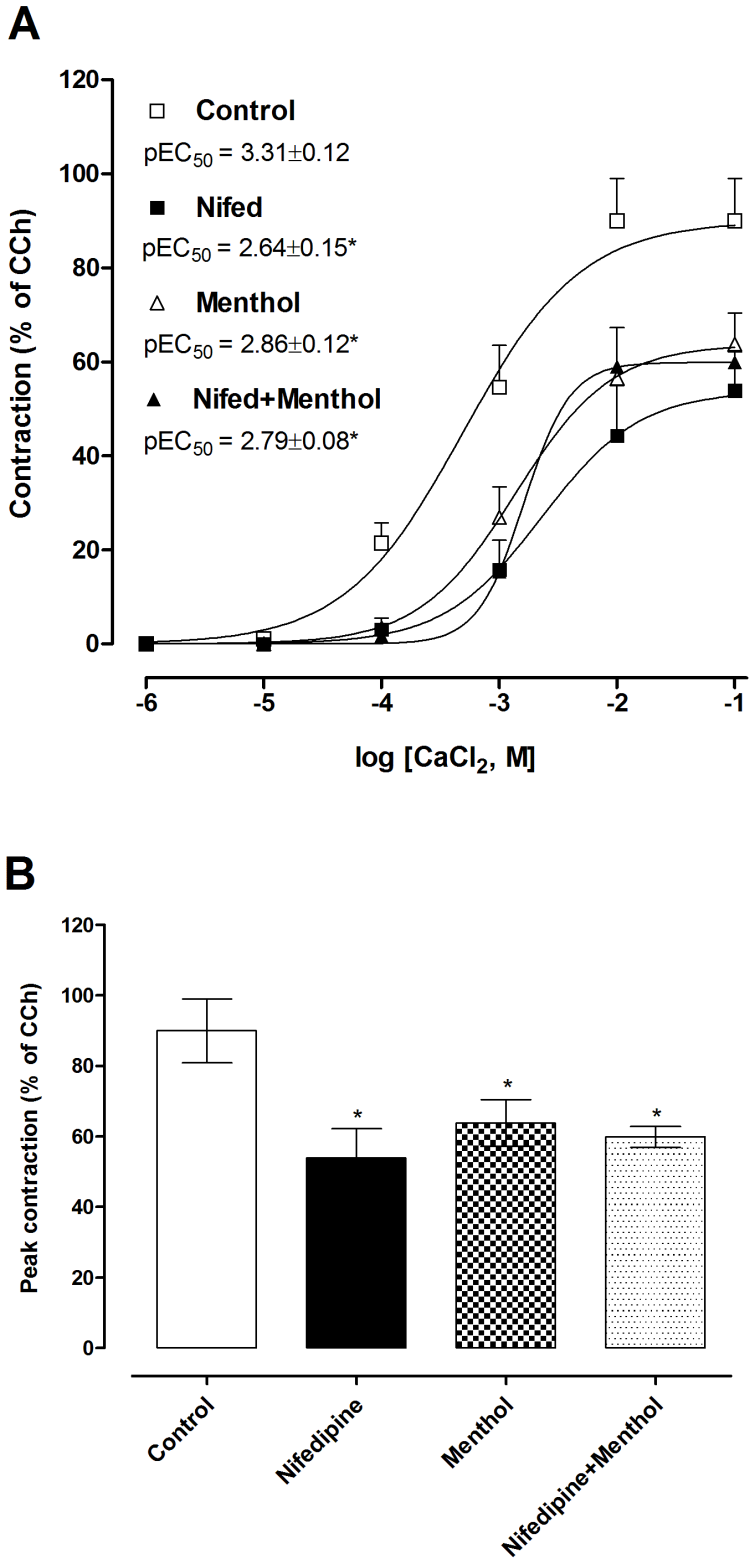
Inhibition of contractions to CaCl_2_ in depolarized bladder strips by menthol and nifedipine. (**A**) Concentration-response curves for contractions to CaCl_2_ (0.01–100 mM) in the presence of nifedipine (1 µM), menthol (300 µM) or both compounds. (**B**) Comparison of maximal responses (E_max_) to CaCl_2_ in the experimental groups. Data represents the mean ± S.E.M. for 3–4 strips in each group. *  =  P<0.05 compared with control (one-way ANOVA followed by Tukey's test).

In bladder strips with intracellular Ca^2+^ stores depleted and depolarized with 80 mM KCl, CaCl_2_ (0.01–100 mM) caused concentration-dependent contractions (Figure 7A). Pre-treatment with nifedipine (1 µM) or menthol (300 µM) shifted pEC_50_ to the right compared with control curve (control: 3.31±0.12, nifedipine: 2.64±0.15 and menthol: 2.86±0.12), and significantly decreased maximal CaCl_2_ contractions (P<0.05; Figure 7B). Pre-treatment with a combination of nifedipine and menthol produced no greater inhibition than either compound alone.

### Menthol inhibited extracellular calcium influx in isolated SMCs

**Figure 8:**
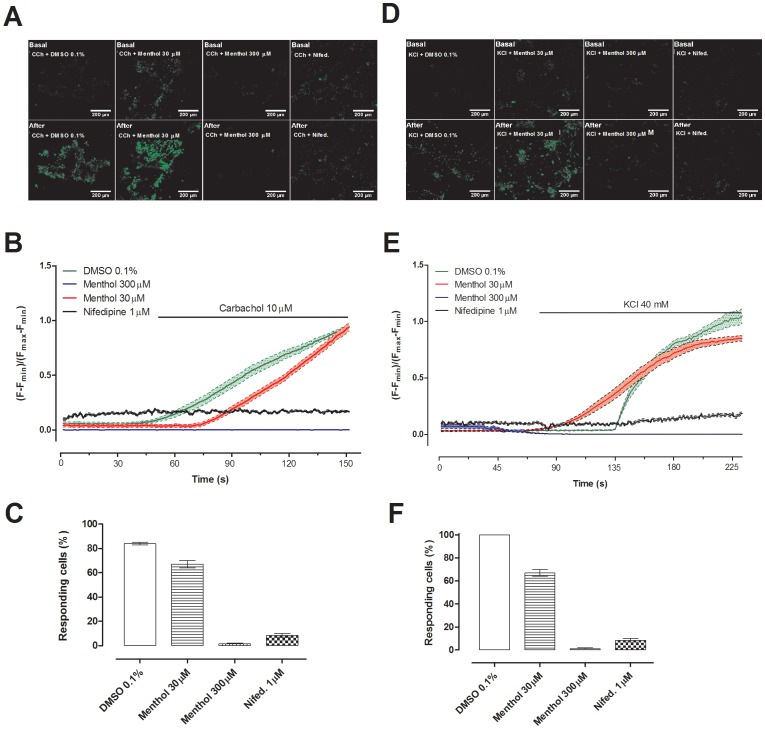
Inhibitory effect of menthol (300 µM) on intracellular Ca^2+^ concentration in bladder smooth muscle cells (SMCs) stimulated with carbachol (CCh; 10 µM) or KCl (40 mM). (A, D) Intensity of emission at 520 nm recorded in SMC's pretreated with DMSO, menthol 30 and 300 µM, or nifedipine (Nifed; 1 µM), before and after carbachol (A) and KCl (D) induces calcium influx. (B, E) Sample trace from one experiment showing mean (F-Fmin)/(Fmax-Fmin) in 30 SMCs exposed to carbachol (B) or KCl (E). Percentage of responding cells to carbachol (C) or KCl (F) stimulation. Data represents the mean ± S.E.M. for 90 cells in total across n = 3 independent experiments.

Primary cultured murine bladder smooth muscle cells were incubated in Ca^2+^-free solution containing CPA (10 µM). Cells were exposed to carbachol (10 µM) in the presence of external Ca^2+^, and an increase in [Ca^2+^]_i_ was observed. A similar increase was observed in cells preincubated for 25 minutes with 30 µM menthol, but preincubation with 300 µM menthol or with 1 µM nifedipine prevented any increase in [Ca^2+^]_i_ (Figure 8A-C). It is notable that cells exposed to 30 µM menthol had a higher baseline [Ca^2+^]_i_ than vehicle-, 300 µM menthol- or nifedipine-treated cells. However the magnitude of the increase in [Ca^2+^]_i_ from baseline was the same as in cells pre-treated with DMSO (Figure 8B). In a separate experiment, smooth muscle cells were depolarized with KCl (40 mM) in the presence of external Ca^2+^. Depolarization induced an increase in [Ca^2+^]i that was unaffected by preincubation with 30 µM menthol, but was abolished by preincubation with 300 µM menthol or 1 µM nifedipine (Figure 8D-F). Again, the cells pre-treated with 30 µM menthol had a higher baseline [Ca^2+^]_i_ than other groups, but increase in [Ca^2+^]_i_ from baseline was similar to that observed in DMSO-treated cells (Figure 8E).

### Menthol increased voiding frequency and decreased peak pressure in intact bladders

**Figure 9:**
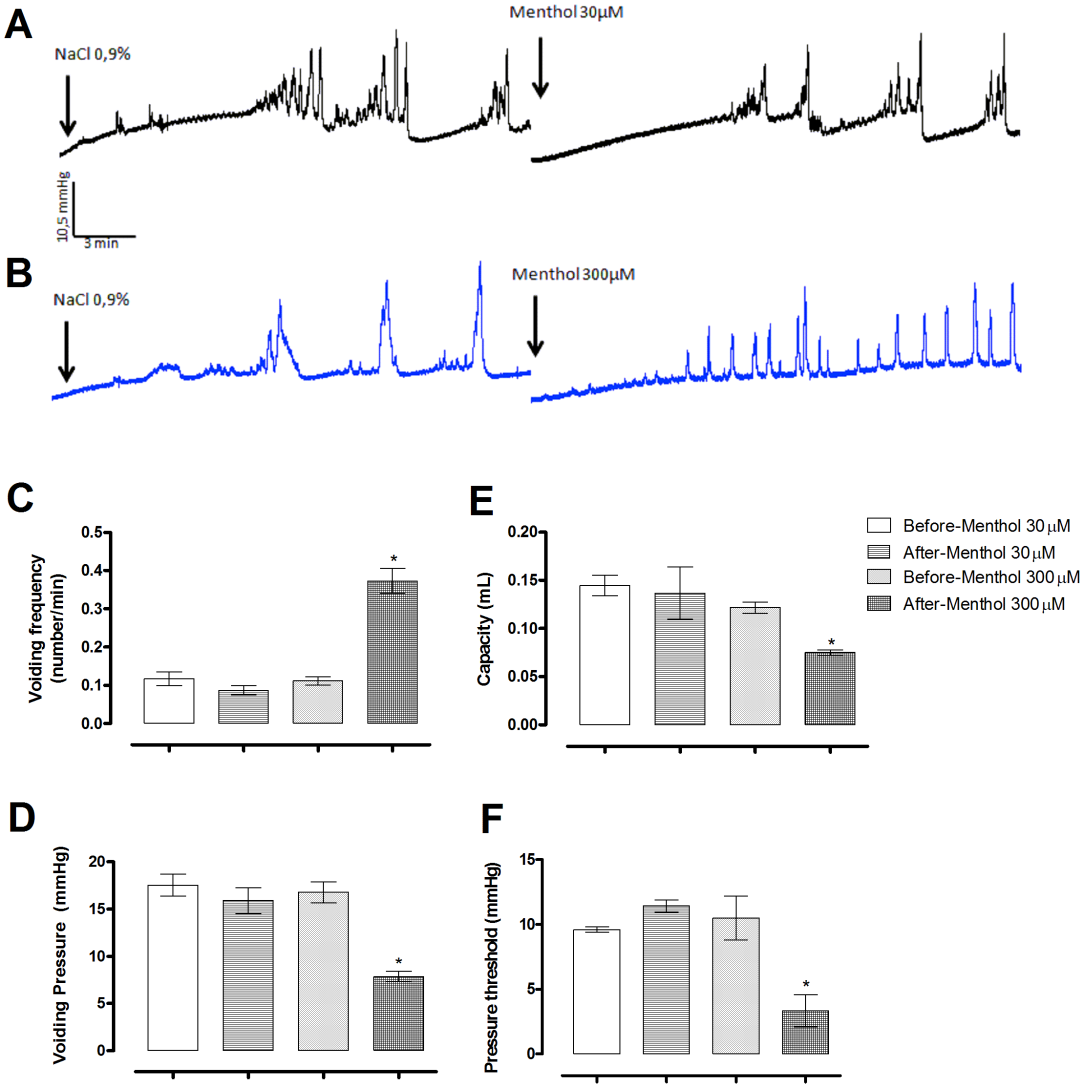
*In vivo* bladder function assessed by cystometry during instillation of menthol 30 µM or 300 µM. (**A, B**) Representative cystometrogram recordings during the instillation of saline (first cystometrogram), menthol 30 µM (**A**) or 300 µM (**B**). Cystometric parameters analysed after treatments: voiding frequency (**C**), voiding pressure (**D**), capacity (**E**) and pressure threshold (**F**). Data represents the mean ± S.E.M. for 3 animals in each group. *  =  P<0.05 compared with saline infusion (one-way ANOVA followed by Tukey's test).

To determine the effects of menthol on bladder function *in vivo*, cystometry was performed on terminally anaesthetised mice. Instillation of 30 µM menthol had no effect on any of the parameters measured. In contrast, instillation of 300 µM menthol significantly increased the voiding frequency, while decreasing the bladder capacity, threshold pressure and peak contraction pressure (Figure 9).

## Discussion

This study is the first to assess the role of TRPM8 in the modulation of bladder contraction *in vitro* to electrical field stimulation or the muscarinic agonist carbachol by menthol. Our results demonstrate that deletion of TRPM8 does not alter muscarinic bladder contractions. Menthol inhibited bladder contractions independently of any activity at TRPM8. This mechanism did not involve blockade of Na^+^ channels or activation of K^+^ channels. Contractions due to extracellular Ca^2+^ entry through voltage-gated Ca^2+^ channels were inhibited similarly by the L-type VOCC inhibitor nifedipine and menthol. Moreover menthol inhibited the increase in [Ca^2+^]_i_ that occurred in cultured SMCs exposed to carbachol or depolarized with KCl. Menthol also exerted inhibitory effects on bladder smooth muscle contraction *in vivo*, decreasing the peak pressure produced during voiding contractions. Altogether our findings suggest that the inhibitory activity of menthol on DSM contractions is not mediated by agonist activity at TRPM8 but instead occurs through inhibition of L-type voltage-gated Ca^2+^ channels.

Electrical field stimulation of bladder strips activates the parasympathetic motor fibres innervating the DSM to release ACh and ATP, causing contraction [21]. The majority of the contraction is due to the activity of ACh on M_3_ muscarinic receptors, and is sensitive to blockade by atropine [22]. Addition of carbachol can also contract bladder smooth muscle through M_3_ receptor activation. EFS and carbachol caused contractions of mouse bladder strips that were unaffected by TRPM8 deletion, suggesting that tonic TRPM8 activity does not contribute to smooth muscle contraction or regulation of contraction in this tissue. Contractions in bladder strips from wild type mice were unaffected by incubation with the TRPM8 agonist icilin, further supporting this conclusion. Instillation of menthol during cystometry experiments caused an increase in the frequency of voiding contractions, similar to that observed in previous studies in the rat and guinea pig [3], [16]. This was probably due to activation of TRPM8 on sensory afferents. However, in contrast to these previous studies, we observed a decrease in the voiding pressure exerted by the bladder in the presence of menthol, whereas intravesical menthol enhanced voiding pressure in the guinea pig [16] and had no effect on pressure in the rat [3]. The reasons for this difference are unclear, but may be related to species differences in the structure of VOCCs. Alternatively the higher concentrations of menthol (600 µM and 3 mM) used in these previous studies may have produced effects at other non-TRPM8 targets to counteract the inhibition of contraction that we observed.

Recent studies have shown that the TRPM8 agonist menthol at concentrations greater than 100 µM inhibited carbachol-induced contraction of rat and pig bladder strips [3], [17]. We observed a similar inhibition of carbachol-induced contractions by 300 µM menthol, along with an inhibition of EFS-induced contractions. Inhibition occurred to the same extent in bladder strips from TRPM8 knockout mice, indicating that it is not dependent on activation of TRPM8 in the bladder strips.

TRPM8 expression in urothelial cells and on bladder sensory afferents has been proposed [12], [13]. Urothelial cells have the ability to sense intravesical changes, responding to chemical, mechanical and thermal stimuli by releasing mediators such as ATP, nitric oxide, and acetylcholine [23]. To further confirm that the inhibitory effects of menthol on muscarinic contractions are not dependent on activation of urothelial TRPM8, we compared carbachol contractions of intact bladder strips with those in strips with the mucosal layer surgically removed. Carbachol caused concentration-dependent contractions in both groups, and menthol inhibited muscarinic contractions in both groups, indicating that menthol's inhibitory effects do not depend on the presence of urothelial cells.

In cultured dorsal root ganglion (DRG) neurons, menthol inhibited voltage-gated Na^+^ channels (TTX-sensitive channels, along with Na_V_1.8 and Na_V_1.9) in a concentration-dependent manner [11]. To determine whether blockade of Na^+^ channels could contribute to the menthol inhibition of muscarinic contractions we removed sodium chloride and sodium bicarbonate and replaced them with equimolar concentrations of NMDG and HEPES. However, muscarinic contractions were unaltered in Na^+^-free solution, indicating that the inhibitory effects of menthol do not occur through inhibition of a sodium conductance in smooth muscle cells or through activation of an action potential to release inhibitory transmitters from bladder neurons. Further support for this conclusion comes from the observation that incubation of bladder strips with the sodium channel inhibitor TTX had no effect on muscarinic contractions or their inhibition by menthol.

Multiple ion channels are expressed by DSM cells and can regulate bladder contraction. Among them, the large conductance voltage- and Ca^2+^-activated K^+^ channels (BK_Ca_ or K_Ca_1.1 channels) are recognised as key regulators of excitability and contractility of the DSM [24]. The SK_Ca_ channels (K_Ca_2.1–K_Ca_2.3) and K_ATP_ channel have also been reported to induce hyperpolarization and to decrease contractibility in urinary bladder smooth muscle from human and guinea-pig [25], [26]. Inhibition of muscarinic contractions by menthol could occur through opening of smooth muscle K^+^ channels to cause K^+^ efflux, hyperpolarisation and subsequent relaxation. However, addition of menthol to bladder strips contracted by exposure to 40 mM KCl caused a concentration-dependent relaxation. The efficacy of menthol in conditions when the concentration gradient for K^+^ efflux is reduced suggests that K^+^ channel activation does not underlie menthol-induced bladder relaxation. Further evidence that menthol acts independently of K^+^ channels is its continuing efficacy in the presence of a cocktail of K^+^ channel inhibitors.

Intracellular Ca^2+^ concentration is controlled by the balance between Ca^2+^ entry and extrusion from the cell. L-type voltage-operated Ca^2+^ channels (VOCCs) are found in the plasma membrane of excitable and non-excitable cells. The opening of these channels generates the upstroke phase of the smooth muscle action potential allowing for net influx of Ca^2+^ to initiate contraction [27]. High levels of extracellular K^+^ depolarize the cell membrane and activate L-type VOCCs, resulting in increased inward movement of Ca^2+^, which causes contraction [27], [28]. Extracellular Ca^2+^ influx through L-type VOCCs has been shown to play an important mechanism in generating the contractile responses to EFS and carbachol in the urinary bladder [27], [29], [30]. In our study, bladder strips treated with CPA to block sarcoplasmic reticulum Ca^2+^ stores and depolarized with KCl were contracted by addition of CaCl_2_ in Ca^2+^-free Krebs' solution. Incubation of the bladder strips with either menthol or nifedipine inhibited this contraction to a similar extent, and the combination of the two compounds did not increase the inhibitory effect.

In cultured SMCs menthol abolished the increase in internal Ca^2+^ concentration produced by carbachol or by KCl depolarization. This was most likely due to a block of VOCCs, as the effects of menthol and nifedipine were the same, preventing further Ca^2+^ influx and allowing the Ca^2+^ efflux and sequestration mechanisms to return the cytoplasmic Ca^2+^ concentration to its low resting level. We noted an elevated baseline [Ca^2+^]_i_ in SMCs pretreated with 30 µM menthol, but this was not apparent in SMCs pretreated with 300 µM menthol, suggesting that it was a quirk of the data rather than an effect of TRPM8 stimulation in these cells. Importantly, the magnitude of the increase in [Ca^2+^]_i_ from baseline following exposure of cells to carbachol or KCL was similar after pre-treatment with either 30 µM menthol or DMSO, indicating that the difference in baseline [Ca^2+^]_i_ did not affect the response.

## Conclusions

Together, our findings indicate that menthol inhibit muscarinic contractions of isolated mouse bladder strips through a mechanism independent of TRPM8 activation, most likely by blockade of L-type VOCCs. More broadly, the identification of menthol actions in the bladder that are independent of TRPM8 activity suggests that other studies linking biological activity of menthol in the bladder to TRPM8 activation must be interpreted with caution, unless a specific effect has been confirmed through the use of knockout mice or receptor antagonists.

## References

[1] Chapple C, Khullar V, Gabriel Z, Dooley JA (2005) The effects of antimuscarinic treatments in overactive bladder: a systematic review and meta-analysis. Eur Urol 48: 5–26. 15885877 10.1016/j.eururo.2005.02.024

[2] Jiang CH, Mazieres L, Lindstrom S (2002) Cold- and menthol-sensitive C afferents of cat urinary bladder. J Physiol 543: 211–220. 12181293 10.1113/jphysiol.2002.019042 PMC2290493

[3] Nomoto Y, Yoshida A, Ikeda S, Kamikawa Y, Harada K, et al (2008) Effect of menthol on detrusor smooth-muscle contraction and the micturition reflex in rats. Urology 72: 701–5. 18336880 10.1016/j.urology.2007.11.137

[4] Lei Z, Ishizuka O, Imamura T, Noguchi W, Yamagishi T, et al (2013) Functional roles of transient receptor potential melastatin 8 (TRPM8) channels in the cold stress-induced detrusor overactivity pathways in conscious rats. Neurourol Urodyn 504: 500–4. 10.1002/nau.22325 23001687

[5] McKemy DD, Neuhausser WM, Julius D (2002) Identification of a cold receptor reveals a general role for TRP channels in thermosensation. Nature 416: 52–8. 11882888 10.1038/nature719

[6] Calixto JB, Kassuya CA, André E, Ferreira J (2005) Contribution of natural products to the discovery of the transient receptor potential (TRP) channels family and their functions. Pharmacol Ther 106(2): 179–208. 15866319 10.1016/j.pharmthera.2004.11.008

[7] Peier AM, Moqrich A, Hergarden AC, Reeve AJ, Andersson D, et al (2002) A TRP channel that senses cold stimuli and menthol. Cell 108: 705–15. 11893340 10.1016/s0092-8674(02)00652-9

[8] Patel T, Ishiuji Y, Yosipovitch G (2007) Menthol: a refreshing look at this ancient compound. J Am Acad Dermatol 57: 873–8. 17498839 10.1016/j.jaad.2007.04.008

[9] Baylie RL, Cheng H, Langton PD, James AF (2010) Inhibition of the cardiac L-type calcium channel current by the TRPM8 agonist, (-)-menthol. J Physiol Pharmacol 61: 543–50. 21081797

[10] Hans M, Wilhelm M, Swandulla D (2012) Menthol suppresses nicotinic acetylcholine receptor functioning in sensory neurons via allosteric modulation. Chem Senses 37(5): 463–9. 22281529 10.1093/chemse/bjr128 PMC3348174

[11] Gaudioso C, Hao J, Martin-Eauclaire MF, Gabriac M, Delmas P (2012) Menthol pain relief through cumulative inactivation of voltage-gated sodium channels. Pain 153: 473–84. 22172548 10.1016/j.pain.2011.11.014

[12] Stein RJ, Santos S, Nagatomi J, Hayashi Y, Minnery BS, et al (2004) Cool (TRPM8) and hot (TRPV1) receptors in the bladder and male genital tract. J Urol 172: 1175–8. 15311065 10.1097/01.ju.0000134880.55119.cf

[13] Mukerji G, Yiangou Y, Corcoran SL, Selmer IS, Smith GD, et al (2006) Cool and menthol receptor TRPM8 in human urinary bladder disorders and clinical correlations. BMC Urol 6: 6. 16519806 10.1186/1471-2490-6-6 PMC1420318

[14] Kullmann FA, Shah MA, Birder LA, De Groat WC (2009) Functional TRP and ASIC-like channels in cultured urothelial cells from the rat. Am J Physiol Renal Physiol 296: F892–F901. 19158342 10.1152/ajprenal.90718.2008 PMC3973644

[15] Everaerts W, Vriens J, Owsianik G, Appendino G, Voets T, et al (2010) Functional characterization of transient receptor potential channels in mouse urothelial cells. Am J Physiol Renal Physiol 298: F692–701. 20015940 10.1152/ajprenal.00599.2009 PMC2838597

[16] Tsukimi Y, Mizuyachi K, Yamasaki T, Niki T, Hayashi F (2005) Cold response of the bladder in guinea pig: involvement of transient receptor potential channel, TRPM8. Urology 65: 406–10. 15708076 10.1016/j.urology.2004.10.006

[17] Vahabi B, Parsons BA, Doran O, Rhodes A, Dean S, et al (2013) TRPM8 agonists modulate contraction of the pig urinary bladder. Can J Physiol Pharmacol 91: 503–9. 23826977 10.1139/cjpp-2012-0406

[18] Lashinger ESR, Steiginga MS, Hieble JP, Leon LA, Gardner SD, et al (2008) AMTB, a TRPM8 channel blocker: evidence in rats for activity in overactive bladder and painful bladder syndrome. Am J Physiol Renal Physiol 0939: 803–810. 10.1152/ajprenal.90269.2008 18562636

[19] Ramos-Filho AC, Mónica FZ, Franco-Penteado CF, Rojas-Moscoso JA, Báu FR, et al (2011) Characterization of the urinary bladder dysfunction in renovascular hypertensive rats. Neurourol Urodyn 30(7): 1392–402. 21661033 10.1002/nau.21074

[20] Mendes-Silverio CB, Leiria LO, Morganti RP, Anhê GF, Marcondes S, et al (2012) Activation of haem-oxidized soluble guanylyl cyclase with BAY 60–2770 in human platelets lead to overstimulation of the cyclic GMP signaling pathway. PLoS One 7(11): 47223. 10.1371/journal.pone.0047223 PMC3493568 23144808

[21] Zimmermann H (2008) ATP and acetylcholine, equal brethren. Neurochem Int 52(4–5): 634–48. 18029057 10.1016/j.neuint.2007.09.004

[22] Tong YC, Hung YC, Lin SN, Cheng JT (1997) Pharmacological characterization of the muscarinic receptor subtypes responsible for the contractile response in the rat urinary bladder. J Auton Pharmacol 17(1): 21–5. 9201556 10.1046/j.1365-2680.1997.00436.x

[23] Birder L, Andersson KE (2013) Urothelial signaling. Physiol Rev 93: 653–80. 23589830 10.1152/physrev.00030.2012 PMC3768101

[24] Petkov GV (2011) Role of potassium ion channels in detrusor smooth muscle function and dysfunction. Nat Rev Urol 9: 30–40. 22158596 10.1038/nrurol.2011.194 PMC3759241

[25] Gopalakrishnan M, Buckner SA, Whiteaker KL, Shieh CC, Molinari EJ, et al (2002) (-)-(9S)-9-(3-Bromo-4-fluorophenyl)-2,3,5,6,7,9-hexahydrothieno[3,2-b]quinolin-8(4H)-one 1,1-dioxide (A-278637): a novel ATP-sensitive potassium channel opener efficacious in suppressing urinary bladder contractions. I. In vitro characterization. J Pharmacol Exp Ther 303(1): 379–86. 12235274 10.1124/jpet.102.034538

[26] Soder RP, Parajuli SP, Hristov KL, Rovner ES, Petkov GV (2013) SK channel-selective opening by SKA-31 induces hyperpolarization and decreases contractility in human urinary bladder smooth muscle. Am J Physiol Regul Integr Comp Physiol 304(2): R155–63. 23174857 10.1152/ajpregu.00363.2012 PMC3543661

[27] Wegener JW, Schulla V, Lee TS, Koller A, Feil S, et al (2004) An essential role of Cav1.2 L-type calcium channel for urinary bladder function. FASEB J 18(10): 1159–61. 15132976 10.1096/fj.04-1516fje

[28] Fry CH, Meng E, Young JS (2010) The physiological function of lower urinary tract smooth muscle. Auton Neurosci 19 154(1–2): 3–13. 10.1016/j.autneu.2009.10.006 19939745

[29] Rivera L, Brading AF (2006) The role of Ca^2+^ influx and intracellular Ca^2+^ release in the muscarinic-mediated contraction of mammalian urinary bladder smooth muscle. BJU Int 98: 868–75. 16978287 10.1111/j.1464-410X.2006.06431.x

[30] Leiria LO, Mónica FZ, Carvalho FD, Claudino MA, Franco-Penteado CF, et al (2011) Functional, morphological and molecular characterization of bladder dysfunction in streptozotocin-induced diabetic mice: evidence of a role for L-type voltage-operated Ca2+ channels. Br J Pharmacol 163(6): 1276–88. 21391978 10.1111/j.1476-5381.2011.01311.x PMC3144540

